# Development and psychometric evaluation of the questionnaire on the contributing factors of tendency towards voluntary single-childedness and childlessness: a mixed method study

**DOI:** 10.1038/s41598-024-51178-5

**Published:** 2024-01-15

**Authors:** Hamid Sharif-Nia, Neda Ahmadzadeh Tori, Fereshteh Behmanesh, Fatemeh Ghaffari, Abolghasem Pourreza

**Affiliations:** 1 Psychosomatic Research Center, Mazandaran University of Medical Sciences, Sari, Iran; 2 Department of Nursing, Amol Faculty of Nursing and Midwifery, Mazandaran University of Medical Sciences, Sari, Iran; 3 Social Determinants of Health (SDH) Research Center, Babol University of Medical Sciences, Babol, Iran; 4 Social Determinants of Health Research Center, Health Research Institute, Babol University of Medical Sciences, Babol, Iran; 5 Nursing Care Research Center, Health Research Institute, Babol University of Medical Sciences, Babol, Iran; 6 Department of Health Education and Promotion, Faculty of Medical Sciences, Tehran University of Medical Sciences, Tehran, Iran

**Keywords:** Health care, Medical research

## Abstract

Couples’ tendency towards voluntary single-childedness and childlessness (VSCC) has turned into a major challenge in all societies and led to different problems such as population aging. A key step to VSCC management is to determine its contributing factors through valid and reliable instruments. This exploratory sequential mixed method study (qualitative-quantitative) was conducted in 2020–2021. Phases of qualitative consists of all couples aged 15–49 in Babol, Iran, who were single or childless. Sampling is based on the purpose, and the number of samples is 20 couples. To collect data, face-to-face and semi-structured interviews were conducted with the participants. Sampling continued until data saturation. The data were analyzed by the conventional content analysis method and quantitative phase. Initially, a qualitative study was conducted on twenty couples, and were analyzed through conventional content analysis. Findings were used to develop QFT-VSCC and then, the face, content, and construct validity as well as reliability were assessed. Construct validity was assessed through exploratory and confirmatory factor analyses and reliability was assessed through internal consistency and stability assessments. The results of the qualitative part analysis consist of 140 codes, 30 primary categories, and nine main categories and two themes (individual limitations and social limitations). The primary QFT-VSCC had 78 items. Fifty-eight items were omitted during validity assessment and the remaining twenty-two items were loaded on five factors during factor analysis. These factors were threatened priorities, inappropriate familial context for childbearing, sense of occupational and social insecurity for the child, social modeling of childlessness, and tendency towards change or stability in marital life. The five factors explained 52.56% of the total variance. All model fit indices in confirmatory factor analysis were acceptable and the Cronbach’s alpha values of QFT-VSCC and all its factors were more than 0.70. The results of convergent validity analysis revealed that all factors had an AVE value greater than 0.5, and the HTMT index for all factors was less than 0.85. This indicated that discriminant validity had been achieved. QFT-VSCC is a simple valid and reliable instrument for VSCC assessment among both men and women.

## Introduction

Fertility is the most principal factor in population changes and has significant role in determining the size, structure, and composition of population in all countries^1^. According to the Organization for Economic Co-operation and Development, the ideal number of children for European countries is 2.2 for men and 2.3 for women^2^. Nonetheless, fertility rate has steadily decreased worldwide in recent decades^1–7^ and fertility rate in most developed and developing countries is currently equal to or even less than population replacement rate^5^. Total fertility rate in Iran started to steadily decrease from 1985 and reached from 6.9 children per woman to 5.5 in 1988, 2.8 in 1996, 2.26 in 1998–2000 (i.e., almost equal to replacement rate), 1.9 in 2006 (i.e., less than replacement rate), and 1.8 in 2011^6^.

Decreased fertility rate is associated with different consequences such as population aging^4^, reduced family size^8^, and will decrease the size of working population^9^.

In addition to the reduction of active and productive population, decreased fertility has great impacts on the elderly population and their social security^10^. Also, as the population ages, various economic, social and psychological challenges appear for older people in society. Entering into old age can increase the use of health care services, due to the widespread need of the elderly for long-term treatment, rehabilitation and care services, which increase costs for their families and the health system. Gonzal and Niplett examined the effects of aging due to increased life expectancy and reduced fertility on capital growth in OECD countries, and concluded that in the political-economic balance, these changes in the demographic structure of society increase taxes, and the share of public investment in social security^11^*.*

Moreover, decreased ferity rate and reduced family size can lead to greater dependence of children on their parents both in childhood and adulthood, senses of loneliness and frustration, and anxiety in adulthood^12^, as well as psychological problems for parents.

Some researchers, in addition to the social problems caused by one-child and childlessness families, including the aging of the population and the reduction of the labor force, have also addressed individual and family issues related to this phenomenon^13^. One of the significant differences between single children and others is their self-centeredness^14^. These children are lonely and incompatible due to the lack of siblings. A study of the psychological characteristics of single children showed that they have more anxiety and depression^15^.

The main factors contributing to decreased fertility rate in recent years are transition from normal pregnancy towards controlled pregnancy^8^ and growing tendency towards voluntary single-childedness and childlessness (VSCC)^5^. Currently, policy makers and sociologists are highly concerned about the public acceptance of VSCC as a postmodern phenomenon^16^ and hence, effective measures are needed for the proper management of this problem and prevention of population aging. A key step to the management of VSCC is to determine its contributing factors^17^. Some previous studies reported that factors such as religious orientation, social media, high marriage age, gender preferences^18^, and increasing tendency towards continuing education^19^ contributed to VSCC.

A study was conducted by Koropeckyj-Cox in the United States to determine the gender gap in attitudes toward infertility. Data were collected by a questionnaire that was collected from 10,648 people over 25 years old. The results of this study showed that women have a more positive attitude towards infertility than men. In women who were less traditional, attitudes toward marriage, gender equality, and employment justified the difference in attitudes toward childlessness^20^. Attitudes toward voluntary childlessness are shaped by a combination of socio-cultural factors and opportunities and constraints^21^.

Careful assessment of the factors contributing to VSCC needs valid and reliable instruments^19^. Examples of the available instruments in this area are Wijma Delivery Expectancy/Experience Questionnaire^22^, the 33-item Father’s Fear of Childbirth Scale^23^, and the Fear of Birth Scale^24^. These instruments measure the tendency towards childlessness among men and hence, are not applicable to women. The Attitude to Fertility and Childbearing Questionnaire^25^ is also appropriate for measuring tendency towards childlessness among women and is not applicable to men. Moreover, previous studies in this area in Iran mostly addressed involuntary single-childedness and childlessness among women and provided no clear data about factors contributing to VSCC among both men and women^25–27^. On the other hand, the contributing factors of VSCC are context-bound and vary from context to context^12^. Therefore, culturally-appropriate instruments are needed in each context for the accurate assessment of the VSCC contributing factors. This study sought to narrow these gaps. The aim of the study is the development and the psychometric evaluation of the Questionnaire on the contributing Factors of Tendency towards VSCC (QFT-VSCC).

## Methods

### Design and setting

This exploratory sequential mixed method study was conducted in 2020-2021in a QFT-VSCC development and a QFT-VSCC psychometric evaluation phase.

### Phase 1. QFT-VSCC development

In this phase, a descriptive qualitative study was conducted and its results were used for item generation. Study setting was rural and urban healthcare settings in Babol, Iran. Babol is the most populated city in Mazandaran province, Iran, and the second most populated city in the north of Iran. Study population consisted of all couples with VSCC in Babol. Participants were twenty couples with VSCC purposively recruited with maximum variation respecting their age, gender, educational level, occupation, and socioeconomic status.

Data were collected through face-to-face semi-structured interviews. Examples of interview questions were “Could you please explain why you decided not to have more children voluntarily?” “What made you decide not to have children anymore?” And "Under what condition do you want to give birth to a child?”, “What factors do you think have led to your wanting one child voluntarily?” “What factors do you think have led to your wanting no child voluntarily?” and “What happened to you after marriage?”.

The interviews were conducted individually in a quiet place that was comfortable for the participant, and with all the factors that provided privacy and psychological security for the participants to freely express their thoughts, feelings and experiences. All interviews were audio recorded using a voice recorder. The length of the interviews was 60–90 min and data collection was continued to reach data saturation. Table 1 shows the demographic characteristics of the participants.

**Table 1:** Demographic characteristic of participants.

Variables	%n
Age	32.76 ± 2.74
Education	
Diploma	10.4
BS	41.5
MS	30.2
Occupation	
Employed	26.3
Unemployed	73.5
Number of children	
Childless	26.30
Only one child	73.70

Data were analyzed concurrently with data collection through conventional content analysis^28^. Initially, each interview was transcribed word by word and the transcript was carefully read. Meaning units were identified and coded and the codes were grouped into subcategories according to their similarities^23^. Subcategories were constantly compared with each other, continuously revised, and grouped with each other to form larger categories. Table 2 shows an example of data analysis. The first author performed data analysis and the coauthors confirmed the accuracy of data analysis.

**Table 2:** An example of data analysis.

Codes	Primary subcategories	Subcategories	Main category
Having no private home and difficulties of living in rented houses and moving to new house Having no car Inability to employ a babysitter High costs of pregnancy-related screening and monitoring tests High costs of living Inability to fulfill children’s needs Children’s high expectations Concern over children’s welfare	Poor financial status	Poor financial status	Financial and occupational problems
	Inability to employ a babysitter		
	Inability to afford living costs		
	Financial dependence on family		
	Inability to provide children with all necessary facilities		
	Inability to fulfill all needs of children		
Unstable employment status Having no fixed job Low job security Negative consequences of a non-ideal job	Unemployment No job security	Insecure job	
Dissatisfaction with spouse’s job Spouse’s shift work	Poor occupational status Negative consequences of having a poor job	Occupational concerns	

Trustworthiness of the qualitative study was ensured through the four criteria of Guba and Lincoln, namely credibility, dependability, confirmability, and transferability^29^. Sampling was performed with maximum variation. Moreover, findings were peer-checked by several external qualitative researchers and member-checked by several participants. All steps of the study were also documented in detail.

### Phase 2. QFT-VSCC psychometric evaluation

In this phase, the psychometric properties of QFT-VSCC, namely face, content, construct, convergent, and discriminant validity as well as reliability were assessed.

In this study, the sample size was determined based on the number of items in the scale multiplied by 10 (20 × 10 = 200) as suggested by Williams et al.^30^.

#### Face validity assessment

The face validity of QFT-VSCC was assessed through qualitative and quantitative methods. In qualitative face validity assessment, ten couples (i.e., twenty individuals) commented on the difficulty, appropriateness, and ambiguity of the items and then, items were revised based on their comments. In quantitative face validity assessment, twenty couples (i.e. forty individuals) were asked to rate item importance on a five-point scale from 1 (“Not important”) to 5 (“Very important”). Then, item impact score was calculated through multiplying the number of participants who scored the item 4 or 5 by item importance score and then, items with impact scores more than 1.5 were considered appropriate^31^.

#### Content validity assessment

The content validity of QFT-VSCC was also assessed through qualitative and quantitative methods. During qualitative content validity assessment, twelve experts in reproductive health, midwifery, sociology, psychology, nursing, religiosity, health education, and economy were invited to comment on item wording, grammar, and allocation and then, items were revised based on their comments. On the other hand, content validity ratio (CVR) and index (CVI) were calculated in quantitative content validity assessment. Accordingly, the same eleven experts were asked to rate item essentiality on a three-point scale as “Not essential” (scored 1), “Useful but not essential” (scored 2), and “Essential” (scored 3). Based on the Lawshe table, items with CVR values lower than 0.59 were excluded. Their rating scores were used to calculate CVR and items with CVR values less than 0.59 were omitted^29^. Moreover, the same experts were asked to rate item relevance and their rating scores were used to calculate item-CVI (I-CVI) and scale-level CVI (S-CVI). Items with I-CVI more than 0.79 were kept for further psychometric evaluation, items with I-CVI between 0.70 and 0.79 were revised, and items were I-CVI less than 0.70 were omitted. S-CVI was also calculated through averaging I-CVIs and an S-CVI value of more than 0.80 was considered acceptable^31^.

#### Item analysis

Before construct validity assessment, fifteen couples (i.e., thirty individuals) completed QFT-VSCC and their data were used for internal consistency assessment. Items with an inter-item correlation coefficient of 0.30–0.80 were considered appropriate and other items were omitted.

#### Construct validity assessment

Construct validity of QFT-VSCC was assessed through exploratory and confirmatory factor analyses.

Exploratory factor analysis.

In this step, 110 eligible couples (i.e., 220 individuals) were purposively recruited from healthcare centers in Babol, to complete QFT-VSCC and a demographic questionnaire. Eligibility criteria were basic literacy skills, no history of infertility, an age of 18–49 years, and agreement for participation. The only exclusion criterion was voluntary withdrawal from the study. The items of the demographic questionnaire were on age, educational level, occupation, and number of children. Sampling adequacy was tested using the Kaiser–Meyer–Olkin (KMO) and the Bartlett’s tests. KMO test values of 0.70–0.80 and 0.80–90 show good and excellent sample size, respectively^32^. Then, exploratory factor analysis was performed to extract the latent factors of QFT-VSCC through maximum likelihood estimation and promax rotation. The minimum acceptable factor loading and communalities value was 0.3 and 0.2 respectively and also the three-indicator rule holds that each factor should have at least three items^33^.

Confirmatory factor analysis.

In this step, 125 eligible couples (i.e., 250 individuals) were selected to complete QFT-VSCC and the demographic questionnaire. Study setting, eligibility criteria, and sampling method in this step were the same as the previous step. Collected data were used to calculate model fit indices, namely incremental fit index (IFI > 0.9), root mean score error of approximation (RMSEA < 0.05), comparative fit index (CFI > 0.9), parsimony comparative fit index (PCFI > 0.5), adjusted goodness of fit index (AGFI > 0.8), parsimony normal fit^34^ .

### Convergent and discriminant validity

The QFT-VSCC was evaluated based on its convergent validity and discriminant validity. Convergence validity requires a Composite Reliability (CR) greater than 0.7 and an Average Variance Extracted (AVE) greater than 0.5. Additionally, in this study, a new criterion was used to assess discriminant validity using the Heterotrait-Monotrait Ratio of Correlations (HTMT) matrix, and all values in the HTMT matrix must be less than 0.85 to achieve discriminat validity^35^.

### Normal distribution, outliers, and missing values

Skewness (± 3) and kurtosis (± 8) values were used to assess univariate normality. Moreover, the Mahalanobis D squared test (P < 0.001) was employed to assess multivariate outliers, the Mardia coefficient of multivariate kurtosis was employed to assess multivariate normality, and missing data were assessed via multiple imputations and were replaced with the mean score^36^.

### Reliability assessment

Cronbach’s alpha was calculated for internal consistency assessment and Cronbach’s alpha values between 0.70 and 0.80 were interpreted as acceptable internal consistency^33^. For stability assessment, ten couples (i.e., twenty individuals) twice completed QFT-VSCC with a two-week interval and then, intraclass correlation coefficient (ICC) was calculated for the test and the retest scores.

### Ethical approval

The Ethics Committee of Tehran University of Medical Sciences, Tehran, Iran, approved this study (code: IR.TUMS.SPH.REC.1399.023). Permissions for the study were also obtained from the Research Administration of this university and provided to the authorities of the study setting. This study was carried out in accordance with the Declaration of Helsinki. Informed consent was obtained from all participants. The participants were also adequately informed about the aim of the study, the confidentiality of their data, and their freedom to refuse participation or withdraw from the study.

## Findings

### Phase 1. QFT-VSCC development

During qualitative, the results of the analysis consist of 140 codes, 30 primary categories, and 9 main categories (livelihood and job problems, unsecured future, threatened priorities, unfavorable family environment for childbearing, fear of becoming a parent, lack of support, Diminished beliefs and attitudes towards childbearing, social role modeling and negative experiences of childbearing) and two themes (individual limitations and social limitations) (Table 3). The first author had prolonged engagement with the study subject matter and attended the study setting for twelve months. Sample quotes of two interviewers follow, related to financial and occupational problems.

**Table 3:** Sub-categories/Categories and Content of the Extracted Themes.

Sub-category	Quotation	Category	Theme
Maintaining appearance and beauty Convenience Social constraints caused by having children Fear of jeopardizing the academic position Fear of jeopardizing the job position	If the child comes, I have to be at home. I can't work anymore or travel or go out	Threatened priorities	Individual limitations
Lack of commitment of couples to each other Failure of one of the spouses to adhere to moral standards Marital conflicts and problems between couples Emotional Divorce	Honestly, after the wedding, I regretted marrying my husband. I wanted to get a divorce. His morals were unbearable. But I had worked so hard to build a life together, and I was dying to leave my life. From the beginning, my husband did not have a good relationship with my family. He didn't even let me go to their house. That's why I wanted to get a divorce in the first nine months. We don't have feelings for each other. We live under the same roof. Having a child in this situation is just destruction	Unfavorable family environment for childbearing	
A negative experience of childbirth A negative experience of pregnancy Unpleasant experiences of the child Limited sexual relations due to childbearing	When I was 9 months pregnant, plus 23 months when I breastfed my baby, we didn't have any sexual relations. We didn't have any. It made me sad. My husband avoided me	Negative experiences of childbearing	
Anxieties of having children Birth phobia	I am afraid that my husband's attention will decrease. Because I saw this in my sister's life. This fear does not allow me to think about having children	Fear of becoming a parent	
Failure to pay attention to religious recommendations Lack of belief in the survival of the generation	I don't believe in religious advice and what does the generation’s survival cost me? If I want to have a child at the age of 35 or not, and then I die at the age of 50, what does it matter if I want to think about these things	Diminished beliefs, and attitudes towards having children	
Inadequate financial condition Inability to meet child needs Career concerns	We cannot support a child. In my opinion, a child is not a priority in life. First, the right living conditions must be provided	Financial and occupational problems	Social limitations
Concern about the child's future Gender-related concerns Problems associated with substance abuse	Now, after several years of living together, we still rent and do not have a house of our own	Unsecured Future	
Lack of social support Lack of family support Lack of government support	My mother is not close to me. There is no one to help me in raising children. I think it is beyond my ability to do all the tasks of raising children alone	Lack of support	
Being influenced by others Influence of media and virtual space	The movies and series that I watch often have a message about the harm that parents suffer because of having a child. The troubles, the loneliness of parents, and the many problems they have to endure for the sake of their children have demotivated me	Social modeling for child avoidance	

The economic issues and financial costs imposed on parents by having children are one of the issues raised by the participants. The facilities that should be provided for the child are effective in the decision-making of individuals.

First participant: “We cannot support a child. In my opinion, a child is not a priority in life. First, the right living conditions must be provided. Now, after several years of living together, we still rent and do not have a house of our own.”

Twenty-seventh participant: “My priority is to have a house first, not children, proper living conditions must be provided.”

**Figure 1:**
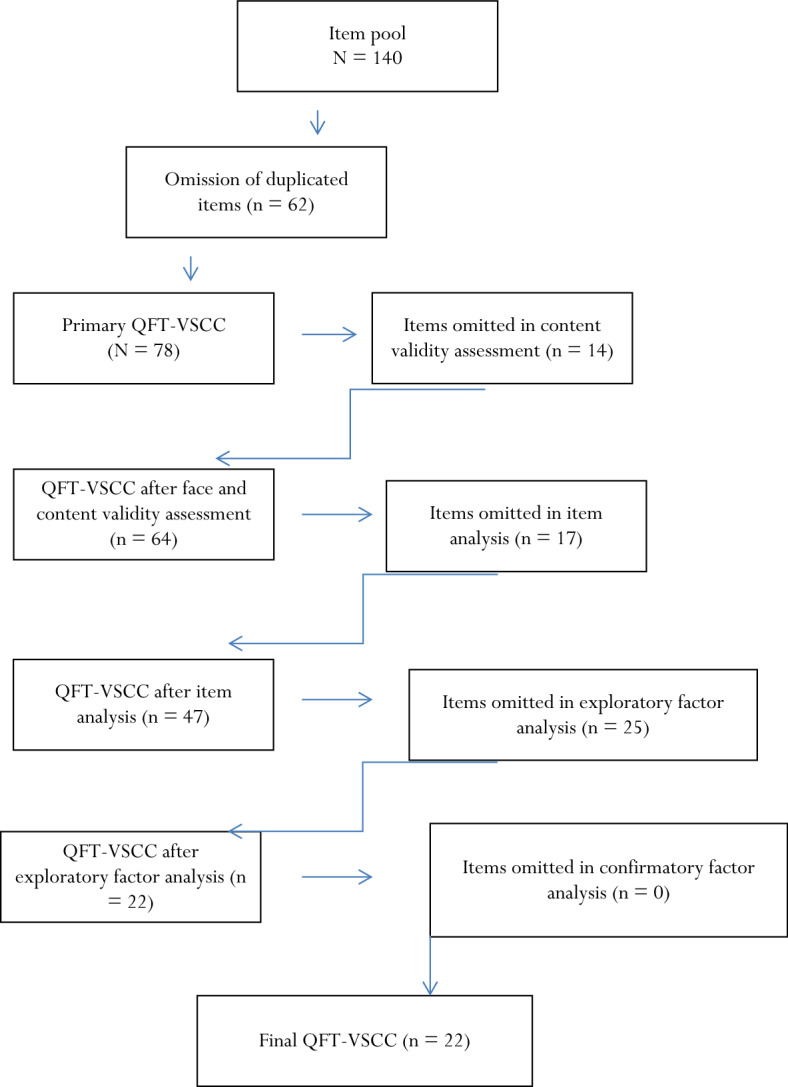
The flow diagram of QFT-VSCC psychometric evaluation.

The 140 codes of the study were used to generate 140 items for QFT-VSCC. Items were compared with each other, similar items were combined, and therefore, the number of items was reduced to 78. Then, the 78-item QFT-VSCC was psychometrically evaluated. (Fig. 1).

### Phase 2. QFT-VSCC psychometric evaluation

#### Face and content validity assessments

Two items were revised in qualitative face validity assessment. In quantitative content validity assessment, fourteen items were omitted due to CVR values of less than 0.59 and all items obtained acceptable CVI values. The S-CVI of the 64-item QFT-VSCC was 0.894.

#### Item analysis

Seventeen items were omitted during internal consistency assessment due to inter-item correlation coefficients of less than 0.30 and the number of items reached 47 (Fig. 1) (Table 4).

**Table 4:** All Items Extracted from Qualitative Phase.

Items	CVR	IIS	kappa
I have a good financial situation	0.66	4.36	0.84
I have the ability to hire a babysitter	0.16	2.63	0.76
I am financially dependent on my family	0.66	3.36	0.76
My/my wife's employment conditions are favorable	0.66	4.54	0.84
I provide all welfare and educational facilities for my child	0.83	4.18	0.92
I am afraid of not being able to raise my child properly	1	4.54	0.92
The lack of security in society makes me unwilling to have children	0.5	3.45	0.92
I am afraid that my child will not be able to grow in society	0.83	3.45	0.92
Improper education in schools makes me unwilling to have children	0.5	3.36	0.66
I am worried that my child will be influenced by others	0.33	3.54	0.41
The uncertainty in the future of my child has caused my unwillingness to have children	0.66	3.81	0.84
I am worried about my child's career and financial future	0.83	4	1
The conditions of society are suitable for having children	0.63	4.63	0.92
I am only interested in having a son or daughter	0.33	2.18	0.66
My own (or my wife's) addiction makes me unwilling to have children	1	4.27	0.84
I have no confidence in continuing to live with an addicted wife	0.83	4.27	0.76
Having children causes the body to become disproportional	0.5	1.45	0.84
Having a child is equivalent to social restrictions	0.33	1.90	0.92
The ease with the adoption rules has made me reluctant to adopt	0.16	1.09	0.41
The freedom to choose surrogacy has made me unwilling to have children	0	1.36	0.54
I can't bear to stay at home to take care of the child	0.33	1.90	0.84
I am afraid of the difficulty of raising a child	0.83	2.90	0.84
Having a child makes me fall behind in life	0.83	1.72	0.84
The child prevents my independence	0.83	1.81	0.84
Having a child makes me not focus on my job	0.83	2.18	1
I am afraid of losing my job position by having a child	1	1.90	0.92
I care more about continuing education than having children	1	2	11
I care more about my job than having children	0.83	1.36	0.92
I am ashamed to become a father/mother at an old/young age	0.33	3.63	0.76
I do not hope for a future life with my wife	0.83	3.27	1
I am afraid that having children will become a factor of forced life with a spouse	1	3.45	0.92
I am afraid that having a child will reduce the intimate/romantic relationship with my wife	0.66	3.63	0.84
I don't want to have children because of my wife's promiscuity	0.81	4.27	1
I am not interested in having children because of my wife's irresponsibility	1	3.90	1
My wife's infidelity has made me unwilling to have children	1	3.18	1
By having children, my wife becomes indifferent to my needs	0.83	2.45	0.92
I don't enjoy my sex with having children	0.33	4.27	0.84
I am satisfied with my married life	0.66	3.72	0.76
I am afraid that my wife's personality will change after having children	0.16	3.54	0.92
I am afraid that my wife will abuse me during pregnancy	0.16	3.54	0.76
I am worried about the interference of others in my child's education	0	4.27	0.76
I am afraid that my child will get genetic diseases	0.83	2.90	0.84
I am worried about being with the child all the time	0.83	4	0.84
I have social support (such as insurance)	1	3.95	0.92
I have the support of my employer during pregnancy	0.83	4.45	0.92
The government has enough support	0.83	4.27	0.84
My wife supports me	0.83	3.81	0.92
I have family support in taking care of my child	0.81	3.81	0.92
I don't want to have children due to a lot of mental conflict	1	3.54	0.92
I don't want to have children due to physical problems	1	3.54	0.92
I don't want to have children due to psychological problems	1	4.27	1
I have the necessary emotional and physical preparation to receive a child	0.81	4.18	1
Living in a stressful environment has made me unwilling to have children	0.83	4.45	0.92
The responsibility of taking care of the people around me has made me unwilling to have children	0.66	2.90	1
I pay attention to religious recommendations regarding having children	0.83	2.72	0.92
The change in my view regarding the role of children in the survival of the generation makes me reluctant to have children	0.53	2.45	0.84
The pattern of filial piety has made me unwilling to have children	0	2.45	0.84
The changing role of children in today's modern family makes me unwilling to have children	0.33	1.90	0.84
Television channels have changed my perspective	0.66	2.27	0.92
My decision to have children is influenced by the views of others/my family	0.83		0.92
Social networks have changed my attitude towards parenting	0.66	2.36	0.92
I have bad memories of giving birth	1	2.81	0.92
I have a negative experience of pregnancy at a young/old age	1	2.36	0.92
I am afraid that the hospital staff do not have enough skills to deliver safely	0.5	2.63	0.84

Abbreviations: CVR: Content Validity Ratio, IIS: Impact score in the face validity.

#### Construct validity assessment

In total, 235 couples (i.e., 470 individuals) completed the 47-item QFT-VSCC and their data were used for construct validity assessment through exploratory factor analysis (n = 220) and confirmatory factor analysis (n = 250). The mean of participants’ age was 32.76 ± 2.74 and 88.9% of them had university degrees, 73.7% of them were employed, 26.30% of them were childless, and 73.70% of them had only one child.

Exploratory factor analysis.

Exploratory factor analysis was performed through maximum likelihood estimation and promax rotation on the data obtained from 220 individuals. The value of the KMO test was 0.86 and the Bartlett’s test was statistically significant (*P* < 0.001) with a test value of 5210.650. Twenty-five items were omitted due to communalities less than 0.2. In parallel analysis criterion, five main factors were extracted and named threatened priorities (six items), inappropriate familial context for childbearing (five items), a sense of occupational and social insecurity for the child (four items), social modeling of childlessness (three items), and tendency towards change or stability in marital life (four items). The eigenvalues of these five factors were respectively 3.394, 2.734, 1.840, 1.694, and 1.907 and these factors explained 52.56% of the total variance of QFT-VSCC (Table 5). The coefficients of internal consistency for the five factors were estimated greater than 0.7. Total Cronbach's alpha was estimated 0.834. The stability was evaluated by ICC = 0.859 and confidence interval 95%.

**Table 5:** The factor structure of QFT-VSCC.

Factors	Factor loading					Eigenvalue (Percentage of variance)	Cronbach's alpha
	1	2	3	4	5		
Factor 1: Threatened priorities							
Q13.Childbearing causes me to lag behind in life Q14.Childbearing is a barrier to my autonomy Q12.Childbearing disturbs my concentration on my work Q15.I will lose my occupational position with childbearing Q26.Instead of addressing my needs, I should allocate all my time to my child Q16.Childbearing threatens continuing my education	.893	−.010	−.039	−.035	.000	3.394 (15.42%)	.874
	.876	−.078	.009	−.016	−.012		
	.860	−.042	.039	−.037	−.040		
	.721	−.082	−.015	.053	.087		
	.612	.096	−.008	.037	.004		
	.442	.160	−.033	.003	−.058		
Factor 2: Inappropriate familial context for childbearing							
Q20.I have no tendency towards childbearing due to my spouse’s immorality	−.074	.988	−.050	−.024	−.037	2.734 (12.42%)	.849
Q22.I have no tendency towards childbearing due to my spouse’s irresponsibility	−.074	.954	−.049	−.036	.015		
Q21.My spouse’s betrayal has made me reluctant towards childbearing	.169	.785	.008	−.029	−.078		
Q34. I am satisfied with my married life	.002	.346	.112	.083	.135		
Q35. I do not hope for a future life with my wife	.083	.330	.137	.122	.126		
Factor 3: A sense of occupational and social insecurity for the child							
Q6. Society is not suitable for rearing my child	.012	.023	.844	−.074	−.047	1.840 (8.36%)	.747
Q7. Ambiguity about my child’s future has made reluctant towards childbearing	−.095	−.039	.822	−.006	−.076		
Q5. I’m worried about my child’s occupational and financial prospect	.011	−.036	.593	−.009	.068		
Q37.Living in a stressful social environment has made me reluctant towards childbearing	.125	.018	.355	.059	.098		
Factor 4: Social modeling of childlessness							
Q44.Media has changed my attitude towards childbearing	−.085	.017	−.010	.952	−.037	1.694 (7.70%)	.756
Q42.My decision about childbearing is influenced by the attitudes of others/my family	−.008	−.009	.021	.696	.004		
Q43.Social networks have changed my attitude towards childbearing	.114	−.046	−.077	.548	−.001		
Factor 5: Tendency towards change or stability in marital life							
Q18.Childbearing reduces my spouse’s attention to me	.009	.005	−.045	−.057	.901	1.970 (8.66%)	.833
Q17.Childbearing prevents me from escaping a forced life	−.078	−.038	.003	.127	.716		
Q19.I fear over the reduction of my intimate relationship with my spouse due to childbearing	.290	.000	−.008	−.071	.573		
Q23.Childbearing makes my spouse indifferent towards my needs	.035	.095	.046	.240	.509		

Total cumulative variance: 52.56%.

To test the construct validity, both convergent validity and discriminant validity were assessed. For convergent validity, composite reliability (CR) should be more than 0.7, and Average Variance Extracted (AVE) should be more than 0.5 for each construct. With respect to discriminant validity, this study used the heterotrait-monotrait ratio (HTMT) of the correlations approach where the HTMT ratio between all constructs should be lower than 0.85^32^. The statistical calculations were performed using SPSS-AMOS27 and JASP0.18.0.0 (Table 6).

**Table 6:** Convergent Validity and Divergent Validity.

Factor	Index			
	CR	AVE	MSV	MaxR(H)
Threatened priorities	0.875	0.545	0.352	0.903
Inappropriate familial context for childbearing	0.846	0.548	0.364	0.926
A sense of occupational and social insecurity for the child	0.777	0.471	0.152	0.803
Social modeling of childlessness	0.772	0.537	0.157	0.832
Tendency towards change or stability in marital life	0.816	0.528	0.364	0.826

Confirmatory factor analysis.

**Figure 2:**
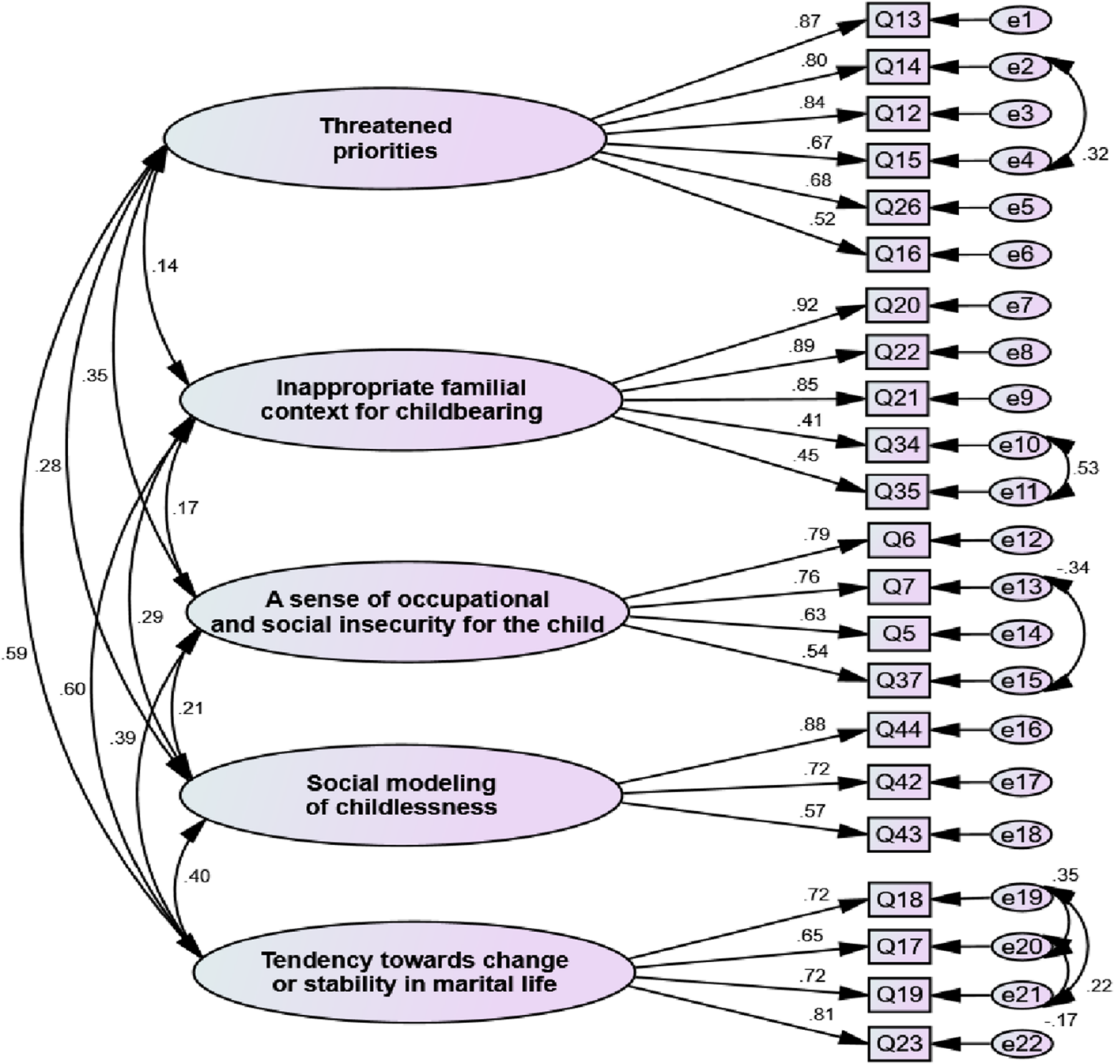
The confirmatory factor analysis model of QFT-VSCC.

The five-factor structure of QFT-VSCC was assessed through confirmatory factor analysis. In the first-order confirmatory factor analysis, the correlations between the measurement errors e1 and e2, e3 and e4, e5 and e6, e7 and e8, e9 and e12, e13 and e14, e15 and e16, e17 and e18, e19 and e20, and e21 and e22 were determined and the value of the Chi-square test was determined to be 287.67 (df = 231; *P* < 0.001). All model fit indices were acceptable (IFI = 0.947, CFI = 0.946, AGFI = 0.816, PNFI = 0.747, PCFI: 0.772, RMSEA = 0.059, CMIN/DF) and the good fit of the model was confirmed. The number of items reached twenty-two at the end of this step (Fig. 2). The factor loading and the Cronbach’s alpha values of all factors were more than 0.5 and 0.70, respectively. The results of convergent validity showed that all factors had an AVE value higher than 0.5, and the HTMT index for all factors was less than 0.85, which indicated discriminant validity.

#### Scoring

The final QFT-VSCC had twenty items in five main subscales. Items were scored on a five-point Likert scale as follows: “Completely disagree”: scored 1; “Disagree”: scored 2; “No idea”: scored 3; “Agree”: scored 4; and “Completely agree”: scored 5. Therefore, the possible total score of the questionnaire is 20–100.

## Discussion

The aim of this study was the development and psychometric evaluation of QFT-VSCC. After psychometric evaluation, the final QFT-VSCC had twenty items in five main subscales, namely threatened priorities, inappropriate familial context for childbearing, a sense of occupational and social insecurity for the child, social modeling of childlessness, and tendency towards change or stability in marital life. These five subscales explained 52.56% of the total variance. The Cronbach’s alpha of QFT-VSCC was 0.86 and the factor loading values of all subscales were more than 0.5. Confirmatory factor analysis confirmed the twenty-item five-factor structure of QFT-VSCC with acceptable model fit indices.

The five subscales of QFT-VSCC can be categorized into the larger category of the personal and social factors to VSCC. Individuals who believe that children are barriers to create ideal conditions have negative attitude towards childbearing^37^. Factors with positive effects on childbearing intention include enjoyment in pregnancy, childbirth, and childrearing, traditional viewpoints, satisfaction with childrearing, sense of survival, and instrumental use of children, while factors with negative effects on childbearing intention are fear over parenting, parenting-related stress, and childrearing challenges^38^. In fact The growth of individualism and the desire for self-actualization has a direct relationship with the couple's fertility goals^39^.

Social factors affecting reproductive behaviors have been expressed in various studies such as: age, education, maternal employment, age of marriage, number of children, age of first pregnancy, access to contraceptives, living conditions, family income, type of acquaintance and traditional marriage, modernity, ethnicity, relationship with husband, female independence in the family, urban or rural, degree of industrialization, social development, ethnic and cultural beliefs and customs, and even society's view of the normal number of family members^5,6,40–43^. In fact, the process of fertility transition is considered in line with developments that have taken place in various economic, social and some traditional aspects of the family and changes in patterns related to marriage and finally, behavior, ideals and childbearing tendencies of people^44^. Global processes such as modernization, industrialization, and urbanization have changed the traditional family structure and the development of new European-type families around the world, as well as making people less focused on their responsibilities to their families and children^45^.

The first subscale of QFT-VSCC was threatened priorities, which explained the highest portion of the variance of QFT-VSCC. The items of this subscale were on the negative effects of children on personal priorities, autonomy, and marital, occupational, and academic achievements. This is in line with the findings of previous studies which reported childbearing as a barrier to the fulfillment of personal wishes^25,46,47^. Another study reported individualism and personal benefits as factors affecting fertility-related behaviors^48^. Moreover, a study showed that tendency towards continuing education had significant relationship with marriage-childbearing interval^49^. Currently, women base their identity more on their social roles than on their maternal, marital, and familial roles.

The second subscale of QFT-VSCC was inappropriate familial context for childbearing which had items on marital conflicts and limited spousal support in childbearing and childrearing. Marital conflicts are mainly attributed to factors such as limited confidence on spouse, poor performance of spouse in marital life, and emotional divorce^18^ and are a major factor affecting childbearing^12,50^. On the other hand, high level of social contribution is associated with greater acceptance of another individual in life^51^.

The third subscale of QFT-VSCC was a sense of occupational and social insecurity for the child which had three items on concerns over child’s occupational and financial prospect, childrearing, and social security. Previous studies also reported concerns over creating a good future for children and concerns over the aggravation of financial problems due to childbirth as barriers to childbearing^16,52^. Consequently, some couples may think that they should never have a child before adequate personal and financial development. Contrary to our findings, a study reported that financial problems had no significant relationship with tendency towards childbearing because high-income families in that study also tended to have fewer children^53^. This contradiction is attributable to the differences between these studies with respect to their settings and their participants’ financial status. Moreover, all participants in the present study were married.

Social modeling of childlessness was the fourth subscale of QFT-VSCC. The three items of this subscale were on the effects of media, social networks, and significant others on the tendency towards VSCC. This is in line with the findings of two former studies which reported that internet and social media have reduced individuals’ tendency towards marriage and childbearing^54,55^. Substantial advances in information and communications technology have significantly changed most aspects of human life and led to the modeling of behaviors of others and the behaviors portrayed in media^12^. However, our findings contradict the findings of a former study which reported that the use of social networks had no significant effects on childbearing tendency^56^. This contradiction is due to the fact that our participants were both men and women aged 18–49 years while that study was conducted only on young women.

The fifth subscale of QFT-VSCC was tendency towards change or stability in marital life with four items on the possible negative effects of childbearing on intimate marital relationships, spouses’ attention to each other, and spouses’ responsiveness to the needs of each other. Similarly, a study reported that childbearing negatively affects marital relationships^25^, while two other studies found that childbearing was associated with better marital relationships^48,55^. This contradiction may be due to differences among these studies regarding their population as well as significant changes in childbearing-related beliefs in recent years.

In comparison with other instruments on VSCC, QFT-VSCC is more appropriate for the careful assessment of VSCC. The Attitude to Fertility and Childbearing Questionnaire has acceptable validity and reliability but is specific to single women^47^. The Attitude towards Fertility and Childbearing Scale was also tested on women with no history of pregnancy in a small geographical area^25^. Moreover, the Childbearing Questionnaire of Miller mainly focuses on the positive and negative childbearing-related preferences and motivations^36^ and the Father’s Fear of Childbirth Scale just assesses fear over childbirth as a factor affecting VSCC tendency and is specific to men^23^.

## Implications

Fertility rate is declining in many countries, and childlessness at all ages is growing in many regions^57^. Recent studies show that among some population groups, the tendency to have one or no child is observed^18^. Statistics show that the tendency to have one or no child is increasing in Iran, and couples prefer to postpone their first pregnancy or remain childless^58^. Some researchers, in addition to the social problems caused by one-childedness and childlessness, including the aging of the population and the reduction of the labor force, have also studied individual and family issues related to this phenomenon^1^.

## Conclusion

In this study, an attempt has been made to examine the issue of voluntary childlessness and factors that reduce the desire to have children.

One of the main reasons for limited studies in this field is the lack of appropriate instruments for use among couples. Therefore, introducing this instrument may pave the way for studies on this subject. A valid and reliable scale could be a good starting point for practitioners to engage with couples.

The results of this study can help to identify the factors affecting the tendency to voluntary childlessness and one-childedness in couples. This may help health policy makers to understand the current situation and help them in comprehensive planning to maintain and increase family health by changing couples' attitudes toward childbearing.

## Strengths and limitations

The strength of the present study was the development and psychometric evaluation of a comprehensive instrument for assessing VSCC tendency among couples. Meanwhile, the study had some limitations. For example, QFT-VSCC was developed as a self-report instrument and psychometrically evaluated in the context of Iran. Therefore, further studies are needed to evaluate the psychometric properties of QFT-VSCC at national level and in other contexts.

## Conclusion

Our study on the contributing factors of tendency towards voluntary single-childness and childlessness adds to existing knowledge by providing a comprehensive questionnaire that can be used to assess the factors that influence individuals' decisions to have or not have children. The study's qualitative analysis identified individual and social limitations as the main themes that affect these decisions. The quantitative analysis identified five factors that explain over 50% of the total variance in these decisions. The study's findings can be used to inform policies and interventions aimed at addressing the factors that contribute to voluntary single-childness and childlessness.

## Data Availability

The datasets used and analyzed during the current study are available from the corresponding author on reasonable request.
